# Paracentesis Cranii in Cases of Hydrocephalus

**Published:** 1882-07

**Authors:** 


### PARACENTESIS CRANII IN CASES OF HYDROCEPHALUS.

Mr. H. P. Dunn believes that: 1. Paracentesis cranii is indicated in all cases of acute and chronic hydrocephalus in which, medicinal treatment having failed, the patient is clearly suffering from the increase of the fluid, and life is threatened.

2. It is the only means by which life can be prolonged, even if by its performance the disease is not arrested.

3. All the fluid which can be obtained should be withdrawn.

4. The operation may be required to be repeated should a re-collection of the fluid be followed by a return of the symptoms which rendered its previous performance necessary.

5. The risks associated with the operation are almost *nil* if carefully performed.—*Lancet,* May 13, 1882.

